# Progression of understanding for the role of Epstein-Barr virus and management of nasopharyngeal carcinoma

**DOI:** 10.1007/s10555-017-9693-x

**Published:** 2017-08-17

**Authors:** Yosuke Nakanishi, Naohiro Wakisaka, Satoru Kondo, Kazuhira Endo, Hisashi Sugimoto, Miyako Hatano, Takayoshi Ueno, Kazuya Ishikawa, Tomokazu Yoshizaki

**Affiliations:** Division of Otolaryngology-Head and Neck Surgery, Graduate School of Medicine, Kanazawa University, 13-1 Takaramachi, Kanazawa, 920-8640 Japan

**Keywords:** Nasopharyngeal carcinoma, Epstein-Barr virus, Alternating chemoradiotherapy, LMP1

## Abstract

Nasopharyngeal carcinoma (NPC) is very common in southern China and Southeast Asia. In regions where NPC is endemic, undifferentiated subtypes constitute most cases and are invariably associated with Epstein-Barr virus (EBV) infection, whereas the differentiated subtype is more common in other parts of the world. Undifferentiated NPC is a unique malignancy with regard to its epidemiology, etiology, and clinical presentation. Clinically, NPC is highly invasive and metastatic, but sensitive to both chemotherapy and radiotherapy (RT). Overall prognosis has dramatically improved over the past three decades because of advances in management, including the improvement of RT technology, the broader application of chemotherapy, and more accurate disease staging. Despite the excellent local control with modern RT, distant failure remains a challenging problem. Advances in molecular technology have helped to elucidate the molecular pathogenesis of NPC. This article reviews the contribution of EBV gene products to NPC pathogenesis and the current management of NPC.

## Introduction

Nasopharyngeal carcinoma (NPC) differs from other head and neck cancers in its epidemiology, pathology, natural history, and response to treatment. Etiologically, Epstein-Barr virus (EBV) is a causative agent in most cases of NPC. Clinically, EBV-associated undifferentiated NPC is highly invasive and metastatic but sensitive to both chemotherapy and radiotherapy (RT) [1, 2]. In contrast, EBV-non-associated differentiated NPC shows similar feature to so call head and neck cancers in that it is locoregionally aggressive rather than highly metastatic and has less chemoradiosensitive properties than EBV-associated NPC [3].

## Epidemiology of NPC

NPC is very common in southern China and Southeast Asia. The recently reported incidence of NPC among men and women in Hong Kong (geographically adjacent to Guangdong province) was 20–30 per 100,000 and 15–20 per 100,000, respectively [4]. An increase in the incidence of NPC has been observed in northern Africa and among the Inuits of Alaska [5]. NPC develops more frequently among the Chinese who have immigrated to other parts of Asia or North America, and it is less common among those born in North America than in those born in southern China [6, 7]. These observations suggest that genetic, ethnic, and environmental factors may play a role in the development of NPC.

## Pathology of NPC

The NPC tumors present with varying degrees of differentiation and have been classified by the World Health Organization (WHO) into three categories [8]. Squamous cell carcinoma, WHO-I tumors, are highly differentiated with characteristic epithelial growth patterns and keratin filaments. Non-keratinizing WHO-II carcinomas retain their epithelial cell shape and growth patterns. Undifferentiated carcinomas, WHO-III, do not produce keratin and lack a distributive growth pattern. Recently, based on an etiological viewpoint, an alternative, simpler classification has been proposed that divides NPC into two histological types, namely squamous cell carcinomas (SCCs) and undifferentiated carcinomas of the nasopharyngeal type (UCNTs) [9]. This classification has been correlated with EBV serology tests.

The association of NPC with EBV was first discovered by seroepidemiological studies, which showed that NPC patients possessed elevated IgA antibodies to EBV replicative antigens [10, 11]. Patients with SCCs had a lower EBV titer, while those with UCNTs had elevated titers. Moreover, EBV-encoded small nuclear RNAs (EBERs), a hallmark of latent EBV infection, were detected in UCNTs but not in SCCs. In North America, tumor histology in 25% of patients was shown to be WHO-I, 12% WHO-II, and 63% WHO-III, while the corresponding histological distribution in southern Chinese patients was 2, 3, and 95%, respectively [12, 13]. Thus, the endemic area of NPC is considered to be implicated in the higher incidence rate of UCNT, namely, EBV-associated NPC. The new classification is more applicable to epidemiological research and has been shown to have a prognostic value. UCNTs have a higher local tumor control rate with therapy, and a higher incidence of distant metastasis [14, 15].

## Biology of EBV

### EBV genes expressed in NPC

**Fig. 1:**
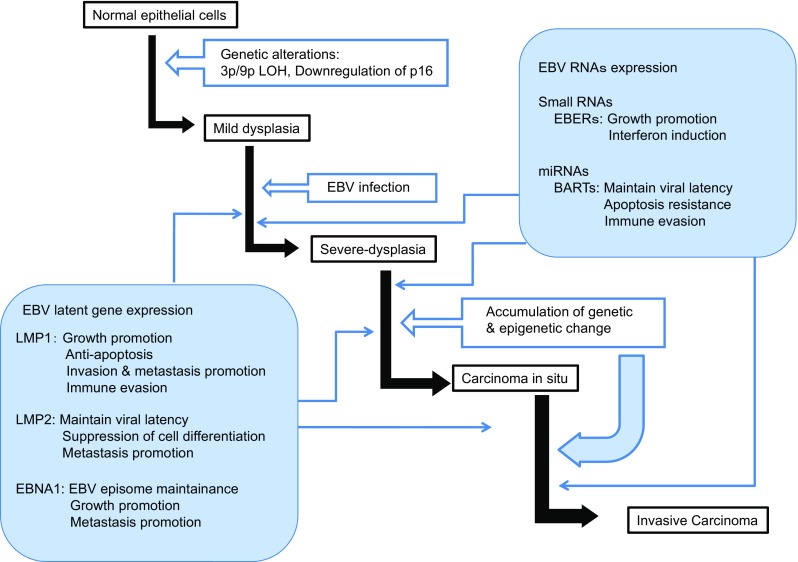
The role of EBV-encoded RNAs and proteins in the development of NPC

EBV latently infects NPC cells and sporadically enters into viral productive lytic infection. Type II latency is maintained, and thus, EBV gene expression is restricted to EBNA1, LMP1, LMP2, EBERs, and BART-encoded miRNAs. Of these genes, LMP1 is a primary oncoprotein encoded by EBV and, therefore, has been enthusiastically studied by many researchers (Fig. 1).

### Tumor initiation, progression, and LMP1

LMP1 has been shown to induce a multitude of effects *in vitro*, including the promotion of cell growth and protection of cells from apoptosis [16, 17]. While these activities contribute to the transforming potential of LMP1, they are dose-dependent. Low levels of LMP1 can induce cell growth and promote cell survival; however, high levels of LMP1 expression are associated with growth inhibition and sensitization to apoptosis in response to serum withdrawal or treatment with TNF, Fas, or chemotherapeutic drugs [18–20]. These paradoxical effects may be associated with the ability of LMP1 to upregulate both pro- and anti-apoptotic genes and disrupt cellular DNA repair programs [21, 22].

### LMP1 promotes cell motility, invasion, and metastasis

The most common clinical symptom of NPC is the presence of cervical lymph node metastasis represented as a neck mass [24]. LMP1 has been shown to contribute to this unique feature of NPC [3]. The first evidence for the relevance of LMP1 regarding the metastatic properties of NPC was that LMP1 induced the matrix metalloproteinase (MMP)-9 [24]. While some studies have identified positive correlations between LMP1 expression and the metastatic status of NPC [25–27], other studies have failed to identify such a link [28]. These conflicting results may be attributable to the sample size and the method used to evaluate LMP1. Further, LMP1 was shown to downregulate cell–cell adhesion and upregulate cell motility *via* the activation of ets-1 and c-Met and the expression of ezrin [29–32]. LMP1 was also shown to induce the expression of Mucin 1 (MUC1), which plays an essential role in tumor invasion and metastasis by opposing cell adhesions [33]. LMP1 affects not only the tumor cell itself but also the degradation of the stroma surrounding the tumor through the upregulation of various MMPs and downregulation of RECK1, an inhibitor of MT1-MMP [24, 34–36]. The induction of MMPs by LMP1 was shown to be mediated by cellular signal transduction systems, such as NF-κB, AP-1, ets-1, and ERK-MAPK [24, 34, 36]. Recently, the cooperation of IL-6 and laminin in LMP1-mediated MMP-9 induction was also reported [37].

### LMP1 promotes lymphangiogenesis

Tumor hypoxia is one of the most common phenomena in human solid cancers. The formation of new blood vessels that supply oxygen and nutrients to the tumor is an important facet of carcinogenesis. Moreover, tumor hypoxia contributes to chemoradiotherapy (CRT) resistance as well as to the malignant tumor phenotype characterized by increased invasiveness and eventually poor prognosis [38]. The overexpression of HIF1α has been related to poor prognosis among patients with NPC [39]. The seven-in-absentia (Siah) protein family consists of human homologs of Siah, a conserved RING finger E3 ubiquitin ligase and an essential downstream component of the *Drosophila* RAS signaling pathway. Siah1 contributes to the stabilization of HIF1α under hypoxic conditions [40]. LMP1 was shown to induce HIF1α through the Siah1-mediated downregulation of prolyl hydroxylases 1 and 3 in nasopharyngeal epithelial cells [41]. The expression of LMP1 significantly correlated with that of Siah1, and both Siah1 and HIF1α-positive cases were shown to have a significantly worse prognosis [42].

Vascular endothelial growth factor (VEGF), which is also closely related with HIF1α, is the other key player in angiogenesis. LMP1 has been shown to induce VEGF production in epithelial cells through the induction of cyclooxygenase 2 (COX-2), FGF-2, MMP-9, and HIF1α [40, 42–46]. However, there is no evidence indicating that aggressive lymphatic metastasis is a common feature of NPC. Moreover, the mechanism by which NPC metastasizes to lymph nodes has not yet been examined, whereas the LMP1-associated upregulation of angiogenic factors has been investigated in detail. A recent study showed that LMP1 promoted lymphangiogenesis through the activation of VEGF-C/VEGF receptor 3 axis, resulting in promotion of lymph node metastasis in NPC [47].

### Genetic and epigenetic alterations and LMP1

Traditionally, the transcription of p16 was shown to be regulated by the retinoblastoma protein (pRb); inactivation of pRb leads to low levels of p16 [48]. However, this is not the case in NPC, as the majority of NPCs exhibit low p16 levels and high pRb levels [49]. NPC cell lines have low levels of p16 secondary to the hypermethylation of p16 [50]. However, this epigenetic alteration may be mediated by the LMP1-induced formation of a c-Jun/Jun heterodimer, which causes the activation of DNA methyltransferase [51]. Additionally, LMP1 was shown to deactivate p16 by inducing the cytoplasmic accumulation of E2F4/5 and ets-2, the nuclear proteins required for normal p16 activity [52]. Homozygous deletion, followed by hypermethylation of the gene, is the most common mechanism of p16 inactivation in head and neck cancer [53].

Comprehensive genome-wide studies revealed not only the loss of p16 but also the multiple loss of heterozygosity of chromosomes 3p, 9p, 11q, 13q, 14q, and 16q in NPC [54, 55]. The deletion of 3p, 9p, and 14q, in particular, was detected in almost all microdissected NPC samples. This finding suggests that the tumor suppressor genes on these chromosomes were inactive [55].

LMP1 may also have many effects on the epigenetic, and eventually, genetic alterations in EBV-infected LMP1-expressing cells. Host genomes appear to be methylated during the course of inactivating viral genomes, including LMP1. Thus, the induction of epigenetic alterations, induced by the existence of EBV, is one of the mechanisms for the promotion of the transformation of EBV-infected nasopharyngeal epithelial cells [56].

### Clinical implication of LMP1 expression in premalignant lesions

The very low incidence of coexisting nasopharyngeal intraepithelial neoplasia with invasive cancer (approximately 3%) and follow-up data indicates the rapid progression of initiated cells through the sequence of dysplasia, carcinoma *in situ*, and invasive cancer [57]. EBV has been detected in all preinvasive carcinoma samples with EBER expression in the majority of the cells. Moreover, the expression of EBNA1, LMP1, LMP2A, and transcripts from BamHI A were detected in all of the premalignant lesions.

A restriction enzyme fragment study showed that preinvasive lesions contained clonal EBV and represented focal cellular growth that developed from a single EBV-infected cell [57]. Generally, metastasis occurs late during the progressive road of malignant tumors. However, LMP1 expression in premalignant lesions enables NPC to metastasize earlier than ordinal cancers, which may be linked to the highly metastatic potential of NPC [3].

## Clinical symptoms and diagnosis

The clinical presentation of NPC is correlated with the extent of primary and nodal disease. Possible routes of primary tumor invasion are anterior spread into the nasal cavity, pterygoid fossa, and maxillary sinuses; lateral involvement beyond the pharyngobasilar fascia into the parapharyngeal and infratemporal spaces; and base of the skull, clivus, and intracranial structures when the disease extends posteriorly and superiorly.

Patients may present a variety of symptoms: non-specific symptoms of epistaxis, unilateral nasal obstruction, auditory complaints, and cranial nerve palsies (cranial nerves third, fifth, sixth, and 12th being the most commonly affected). After analyzing 525 Japanese NPC patients, the symptoms were a neck mass in 52% of the patients, ear symptoms in 48%, nasal symptoms in 27%, headaches in 10%, pharyngeal symptoms in 9%, ophthalmologic symptoms in 9%, and cranial nerve symptoms in 9%. For cranial nerve symptoms, the abducens nerve (cranial nerve VI) is the most commonly impaired nerve, followed by the trigeminal nerve (V) [23].

Thorough pretreatment assessment by endoscopy, magnetic resonance imaging (MRI), and positron emission tomography (PET) coupled with computed tomography (CT) is fundamental. Biopsy samples are obtained for pathological diagnosis.

Characteristic histological features constitute the microscopic morphology of NPC, but at times, distinguishing between the undifferentiated subtype and lymphoma might be difficult. In such instances, immunohistochemical markers specific to individual tumor types (leucocyte common antigen, lymphoma; S100, melanoma; MNF116, a pancytokeratin marker) and *in situ* hybridization to EBERs can supplement hematoxylin and eosin staining. Other useful investigations for the confirmation of a diagnosis of NPC are quantitative assessments of plasma immunoserology and EBV-DNA [58, 59].

MRI provides better resolution than CT in terms of assessing parapharyngeal spaces, marrow infiltration of the skull base, intracranial disease, and deep cervical nodes. 18F-fluorodeoxyglucose (18F-FDG)-PET is sensitive and accurate in the detection of nodal metastasis but lacks the soft tissue resolution of MRI for the assessment of the primary tumor [60, 61]. In terms of distant metastasis staging, several studies have concluded that 18F-FDG-PET is substantially more sensitive and accurate than the conventional work-up consisting of chest radiography, abdominal ultrasound, and skeletal scintigraphy [62, 63]. One of the unique clinical features of NPC is the propensity for distant metastasis. At the time of presentation, 5–11% of patients have distant metastasis [64]. During the course of the disease, 50–60% of patients develop distant metastases [65]. Thus, MRI and 18F-FDG-PET are recommended as the preferred modalities for staging in patients with TNM stage III, IVA, or IVB NPC. Several studies reported that 18F-FDG-PET uptake measured by SUV (max) or total lesion glycolysis predicted overall survival [66, 67]. However, in terms of predicting the treatment outcome in NPC patients, 18F-FDG-PET was not sufficiently accurate to be clinically acceptable.

## Staging and prognosis

The tumor, nodes, and metastases (TNM) staging is the most important prognostic factor for NPCs [68]. Among the changes in the eight edition of the TNM staging system (2016), the medial pterygoid muscle and/or lateral pterygoid muscle involvement was changed from T4 to T2, prevertebral muscle involvement was added as T2, the supraclavicular fossa was replaced with the lower neck, and this was merged with a maximum nodal diameter > 6 cm as N3, and T4, and N3 were merged as stage IVA criteria. These changes not only will lead to a better distinction of risk but also will optimize the balance in clinical practicability and global applicability [69]. A study reported in 1992 showed that the tumor’s WHO histological type and the RT dosage and coverage were also significant independent prognostic factors [70]. The histological type, WHO-I, which is frequently seen among the Caucasian population, was found to be associated with adverse prognosis [71].

Indeed, most other known prognostic factors are directly or indirectly related to the extent or bulk of the tumor. A large variation of tumor volume is present in T stages, and primary tumor volume represents an independent prognostic factor of local control. Its validity has been confirmed in patients with T3 and T4 tumors, and there is an estimated 1% increase in the risk of local failure for every 1-cm^3^ increase in primary tumor volume [72]. Guo et al. suggested an improved predictive ability of T classifications when primary tumor volume is incorporated [73]. Although the incorporation of tumor volume into TNM classification is attractive, there are still important issues to be addressed. Measurement of tumor volume can be affected by imaging modalities, measuring protocols, inter-observer and intra-observer variability [74, 75].

The amount of circulating EBV-DNA in NPC patient is estimated to reflect the tumor load and is positively correlated with disease stage. Further, it has been shown to have prognostic importance [76]. Especially, either plasma or serum EBV-DNA titer is estimated to reflect tumor volume. Biologically, such EBV-DNA reflects reproduced or released DNA from dead or dying tumor cells. On the other hand, EBV-specific DNA released as exosome may reflect the biological feature of the alive NPC tumor cell [77].

## Treatment

### Radiation therapy

Most of the NPC cases have wild-type p53, which is highly radiosensitive. Thus, RT plays a central role in the treatment of all stages of NPC patients without distant metastases. A good locoregional control should be the prime objective of treatment, as locoregional relapses represent a significant risk factor for the development of distant metastases [78]. Conventional 2-dimensional (2D) RT successfully controlled between 75 and 90% of patients with T1 and T2 tumors and 50–75% of T3 and T4 tumors [79, 80]. Nodal control is achieved in 90% for N0 and N1 cases, but the control rate drops to 70% for N2 and N3 cases [79]. Because interruptions and prolonged treatment adversely affect outcome in RT for NPC, every effort should be made to maintain the treatment schedule [81].

Because of the high incidence of occult neck node involvement, prophylactic neck radiation in node-negative patients was usually recommended [82]. It has long been documented that NPC metastases to the cervical lymph nodes follow an orderly pattern, from the upper neck inferiorly to the lower neck and then the supraclavicular fossa. Recent randomized studies have shown that the survival and tumor control rates in node-negative patients did not significantly differ between the selective neck irradiation group confined to levels II, III, and VA and the whole neck irradiation group [83]. This avoidance of the lower neck and level IB is useful because by the latter, the submandibular gland is spared leading to a reduced risk of xerostomia.

RT for NPC is challenging because the nasopharynx is anatomically surrounded by an array of radiosensitive structures such as the brain stem, spinal cord, pituitary-hypothalamic axis, temporal lobes, eyes, middle, and inner ears, and parotid glands. As NPCs tend to infiltrate and spread to normal organs, the irradiation target volumes in NPC are very irregular. For patients with early disease, as they have a good chance of survival, radiation toxicities in these even non-critical structures would affect the quality of life of the survivors. However, for patients with locally advanced disease such as skull base or intracranial extension, the challenge lies in achieving an adequate tumor control to spare the critical organs.

The major limitations of conventional 2D RT for NPC can now be overcome with three-dimensional (3D) conformal RT and intensity-modulated radiation therapy (IMRT) [84, 85]. IMRT is an advanced form of 3D conformal RT, in which a high dose is irradiated to the tumor while irradiating a low dose to normal tissues. Such ability of IMRT to deliver a more conformal radiation dose to the target area and spare surrounding structures seems to decrease the toxicity of CRT [86].

Some researchers reported excellent local control as more than 90% of NPC patients achieved local control with IMRT [87], even in cases of advanced T3–4 diseases [88]. It has also been shown that preservation of salivary function and quality of life improves for IMRT survivors [89, 90]. A recent multi-center study also showed that in a multi-institutional setting, it was possible to achieve 90% local control rate excellence with IMRT as reported in single institutions [91]. Thus, IMRT has gradually been considered as the new standard RT for NPC.

### Chemotherapy

Radiosensitivity correlates well with chemosensitivity; thus, NPC is also chemosensitive. Many clinical studies investigated the advantages of chemotherapy for NPC. A pivotal study was reported by the Head and Neck Intergroup in 1998, using concurrent RT with cisplatin (100 mg/m^2^ days 1, 22, 43) followed by adjuvant cisplatin and 5-fluouracil (5-FU) (cisplatin 80 mg/m^2^ day 1 and 5-FU 1000 mg/m^2^/day, days 1–4, 4-week cycles for 3 cycles) [92]. Compared with RT alone, CRT significantly improved progression-free survival and overall survival. The Intergroup then conducted other (0099) randomized trials, using a similar design, in endemic regions in Asia, to validate the Intergroup results. Three randomized trials were subsequently reported from Hong Kong [93], Singapore [94], and China [95], respectively.

The advantage of neoadjuvant chemotherapy (NAC) has not been established in combination with RT alone [96, 97]. Furthermore, the role of NAC in combination with concurrent CRT (CCRT) is yet to be confirmed. In a phase 3, multi-center, randomized controlled trial, the addition of NAC with docetaxel, cisplatin, and 5 FU (TPF) to CCRT significantly improved failure-free survival in advanced NPC [98]. The patients with previously untreated, stage III–IVB (except T3–4 N0) NPC were enrolled. Eligible patients were randomly assigned, 241 patients were assigned to NAC plus CCRT, and 239 to CCRT alone (three cycles of 100-mg/m^2^ cisplatin every 3 weeks, concurrently with IMRT). NAC was three cycles of intravenous docetaxel (60 mg/m^2^ on day 1), intravenous cisplatin (60 mg/m^2^ on day 1), and continuous intravenous fluorouracil (600 mg/m^2^ per day from day 1 to day 5) every 3 weeks before CCRT. After a median follow-up of 45 months (IQR 38–49), 3-year failure-free survival was 80% (95% CI 75–85) in the NAC plus CCRT group and 72% (66–78) in the CCRT alone group (hazard ratio 0.68, 95% CI 0.48–0.97; *p* = 0·034).

TPF may play an important role to improve the treatment results of NPC. Kong et al. treated 52 patients with stage III NPC NAC + CCRT and 64 patients with non-metastatic stage IV NPC. All patients received TPF (docetaxel 75 mg/m^2^, cisplatin 75 mg/m^2^, and 5-FU 2500 mg/m^2^ every 3 weeks for three cycles), followed by cisplatin 40 mg/m^2^ per week concurrently with either 3D conformal RT or IMRT. With a median follow-up of 32.9 months, the 3-year overall survival rates were 94.8 (95% CI, 87.6–100%) and 90.2% (95% CI, 81.8–98.6%) for the stage III group and the IVA/IVB group, respectively. The 3-year progression-free survival, distant metastasis-free survival, and local progression-free survival rates were 78.2 (95% CI, 64.6–91.8%), 90.5 (95% CI, 79.7–100%), and 93.9% (87.1–100%), respectively, for stage III group and 85.1 (95% CI, 75.1–95.1%), 88 (95% CI, 78.6–97.4%), and 100%, respectively, for stage IVA/IVB group. Grade 3/4 neutropenia was observed in 64 patients (55.2%) and nausea and vomiting for 23 patients (19.8%) [99]. Similarly, taxane-based NAC was expected to have a positive effect on the control of metastatic disease [100, 101]. Moreover, for patients with intracranial invasion, replanning the delineation for tumor volume after NAC improved the local disease control and reduced IMRT associated adverse events. This meta-analysis compared the overall survival, locoregional failure, and distant metastasis-free survival between NAC + CCRT and CCRT. In the meta-analysis, the three treatments with the highest probability of benefit on overall survival were the addition of adjuvant chemotherapy (AC) to CRT, followed by CRT and the addition of NAC to CRT, with respective hazard ratios (HRs [95% CIs]) compared with RT alone of 0.65 (0.56 to 0.75), 0.77 (0.64 to 0.92), and 0.81 (0.63 to 1.04). The addition of AC to CRT achieved the highest survival benefit and consistent improvement for all end points. The addition of NAC to CRT achieved the highest effect on distant control. A sufficient amount of an anti-cancer agent is required to control the distant metastasis [102]. However, at present, it is unclear whether the addition of NAC to CCRT improves survival rates compared with CCRT (Table 1) [103–105].

**Table 1:** Randomized trials of chemoradiotherapy with or without neoadjuvant chemotherapy

Author	Year	Group	Radiotherapy	Chemotherapy regimen	NAC regimen	No. of patients	Overall survival		Progression-free survival		Ref.
							Control *vs* experimental	HR (95% CI); *p* value	Control *vs* experimental	HR (95% CI); *p* value	
Hui et al.	2009	CCRT NAC + CCRT	2 Gy/F × 5F/week total dose: 66 Gy	Cisplatin 40 mg/m^2^ × 8	Docetaxel 75 mg/m^2^, cisplatin 75 mg/m^2^ every 3 weeks × 2	65	3-Year OS 67.7 *vs* 94.1%	0.24 (0.078–0.73); *p* = 0.012	3-Year PFS 59.5 *vs* 88.2%	0.49 (0.20–1.19); *p* = 0.12	[100]
Fountzilas et al.	2012	CCRT NAC + CCRT	Total dose: 66–70 Gy	Cisplatin 40 mg/m^2^ × 7	Cisplatin 75 mg/m^2^, epirubicin 75 mg/m^2^, paclitacel 175 mg/m^2^ every 3 weeks × 3	141	3-Year OS 71.8 *vs* 66.6%	0.95 (0.48–1.89); *p* = 0.888	3-Year PFS. 63.5 *vs* 64.5%	1.40 (0.71–2.77); *p* = 0.334	[104]
Tan et al.	2015	CCRT NAC + CCRT	Total dose: 70 Gy	Cisplatin 40 mg/m^2^ × 8	Gemcitabine 2000 mg/m^2^, carboplatin AUC 5 m^2^, paclitaxel 140 mg/m^2^ every 3 weeks × 3	172	3-Year OS 92.3 *vs* 94.3%	1.05 (0–2.19); *p* = 0.494	3-Year DFS 67.4 *vs* 74.9%	0.77 (0.44–1.35); *p* = 0.362	[105]
Sun et al.	2016	CCRT NAC + CCRT	Total dose:66 Gy	Cisplatin 100 mg/m^2^ × 3	Docetaxel 60 mg/m^2^, cisplatin 60 mg/m^2^, fluorouracil 1200 mg/m^2^ every 3 weeks × 3	480	3-Year OS 86 *vs* 92%	0.59 (0.36–0.95); *p* = 0.029	3-Year FFS 72 *vs* 80%	0.68 (0.48–0.97); *p* = 0.034	[98]
Ma et al.	2001	RT NAC + RT	2Gy/F × 5F/week total dose 72 Gy	–	Cisplatin 100 mg/m^2^, bleomycin 20 mg/m^2^, 5-FU 4000 mg/m^2^ every 3 weeks × 2 to 3	64	5-Year OS 56 *vs* 63%	Not tested; *p* = 0.11	5-Year RFS 49% *vs* 59%	Not tested; *p* = 0.05	[97]

Abbreviations used: *CCRT* concurrent chemoradiotherapy, *RT* radiotherapy, *NAC* neoadjuvant chemotherapy, *OS* overall survival, *PFS* progression-free survival, *DFS* disease-free survival, *FFS* failure-free survival, *RFS* relapse-free survival, *Ref.* references

The role of AC in NPC has been reviewed negatively. To date, no randomized phase III studies have demonstrated a survival advantage of AC. In the meta-analysis, the magnitude of the overall survival benefit observed in the subgroup receiving CRT plus AC (*n* = 1267; HR, 0.65; 95% CI, 0.56–0.76) seemed to be larger than that in the CRT-alone subgroup (*n* = 1834; HR, 0.80; 95% CI, 0.70–0.93) [106]. In the largest phase III study reported to date, Chen et al. aimed to assess the contribution of AC to CCRT *vs.* CCRT alone. They conducted an open-label phase 3 multi-center randomized controlled trial for the patients with non-metastatic stage III or IV (except T3–4N0) NPC at seven institutions in China. Patients were randomly assigned to the CCRT plus AC group (*n* = 251) and to the CCRT alone group (*n* = 250). Patients in both groups received 40-mg/m^2^ cisplatin weekly up to 7 weeks, concurrently with RT. The CCRT plus AC group subsequently received 80 mg/m^2^ adjuvant cisplatin and 800 mg/m^2^ per day fluorouracil every 4 weeks for three cycles. The estimated 2-year failure-free survival rate was 86% (95% CI 81–90) in the CCRT plus AC group and 84% (78–88) in CCRT only group (HR 0.74, 95% CI 0.49–1.10; *p* = 0.13). In this series, adjuvant cisplatin and fluorouracil chemotherapy did not significantly improve failure-free survival after CCRT in locoregionally advanced NPC. Furthermore, compliance was a problem. Only 63% of patients could complete the planned chemotherapy. They concluded that longer follow-up was needed to fully assess survival and late toxic effects, but such regimens should not, at present, be used outside well-designed clinical trials [107].

Among the molecular markers, the most studied is plasma EBV-DNA, which is universally associated with the non-keratinizing subtype of NPC. EBV-DNA is not only a good prognosticator, but it is also useful for assessing treatment response and detecting disease relapse. Levels of post-treatment plasma EBV-DNA in patients with NPC appear to strongly predict progression-free survival and overall survival. Additionally, this is accurate and to accurately reflect the post-treatment residual tumor load. The current research focuses on the effect of the selected patients that probably referred to maximal benefit from AC. It was further suggested that high circulating plasma EBV-DNA loads of 500 copies per mL. Similarly, the EBV-DNA tested at 6 weeks post-primary treatment can predict the probability of subsequent relapse of NPC [108]. This study led to the adoption of post-treatment plasma EBV-DNA load as a prognostic marker. Further studies are needed to investigate the utility of post-treatment plasma EBV-DNA in individualizing AC.

Although some reports resulted in an unfavorable feasibility, it is now generally accepted that Intergroup 0099 CCRT-AC protocol is feasible for NPC, including in endemic and non-endemic areas. The 0099 study showed that CRT was the most efficacious NPC treatment. Three randomized trials that evaluated CCRT-AC showed negative results [109–111]. Thus, there is still debate on the efficacy of AC in the Intergroup 0099.

Compared to the Intergroup 0099 Trial, alternating CRT in a multi-center prospective study in Japan was characterized by a decreased total dose of CDDP (540 mg/m^2^ in the Intergroup 0099 *vs.* 300 mg/m^2^ in Japan), a shorter treatment period (130 days in the Intergroup 0099 *vs.* 83 days in Japan), a higher treatment completion rate (55%: 43 of 78 cases in the Intergroup 0099 *vs.* 80%: 70 of 87 cases in Japan), and a better 3-year overall survival (78% in the Intergroup 0099 *vs.* 92% in Japan) [112]. The overall survival and progression-free survival at 5 years were 78.04 (95% CI 69.1–87.0%) and 68.74% (95% CI 58.8–78.7%), respectively [113]. The long-term outcomes of alternating CRT for NPC were thought to be promising. However, patients who do not receive chemotherapy completely have a good prognosis. A sufficient dose of CDDP and 5-FU is at least more than 200 and 8000 mg/m^2^, respectively [114]. Although the final therapeutic value of this alternating CRT cannot be currently evaluated, this method can be used in a controlled clinical trial in the future to compare therapeutic results with those of the concurrent CRT.

### Management of residual or recurrent disease

Although concomitant CRT generated remarkable improvement in the management of NPC, some patients still developed locoregional recurrence presenting as persistent or recurrent tumor. Early detection is essential for any form of salvage therapy to be successful. Salvage surgery for locally recurrent NPC is warranted, especially when the disease is confined to the nasopharynx [115]. Even for patients with synchronous locoregional failures, salvage surgery should be considered for selected patients. There are three popular approaches for the nasopharynx, the transoral (transpalatal), transmaxillar (maxillar swing), and endoscopic approaches. Patients whose local disease was treated by surgical resection had a 3-year local control rate of 71% compared to 38% in those who received reirradiation using brachytherapy or external RT. For regional disease, the 3-year nodal control rate after radical neck dissection was 65% compared with 24% by reirradiation [116]. In all the cases, repeat irradiation has to be administered with utmost care given the risk of fatal late effects. Reports of patients who were salvaged with IMRT attributed half of all mortality to late effects [117, 118].

Isolated neck failure occurs in less than 10% of patients with contemporary treatment [119]. In the unlikely event of occurrence, surgical neck dissection is the preferred choice for salvage, and it is even effective for deep retropharyngeal nodal metastasis [120]. A residual or recurrent tumor in the cervical lymph nodes after RT is notoriously difficult to confirm, as in some lymph nodes only clusters of tumor cells are present [121]. Thus, sometimes the diagnosis can only be confirmed after salvage surgery.

The other argument is the type of dissection. Wei et al. analyzed serial sections in a series of whole-neck dissection specimens and found that all levels of the neck compartment had the potential of being involved. Level II was the most common (53%). They also found the incidence of extranodal extension to be 84% for patients with extensive recurrent or persistent neck disease. The analysis of their results, however, indicates that metastases at level I or V occurred in only 4% of patients [121]. Yen et al. have reported a high 5-year OS with the salvage dissection, which in many patients included levels II–V only. They also found that musculature or nerve involvement at level V or extracapsular spread of the tumor was associated with a decreased survival [122]. Radical neck dissection has been indicated as the standard treatment for recurrent or persistent NPC of the neck. Considering that sublevel IA is not usually involved in NPC, the dissection of this sublevel in these tumors may be considered unnecessary.

Despite the varying degrees in the success of surgery or reirradiation in salvage therapy for highly selected patients with local recurrence, the vast majority of patients with recurrent disease are only amenable to palliative chemotherapy. In phase II studies of platinum-based doublets that are in popular use today, the median overall survival rates in the first-line setting ranged from a minimum of 11 to a maximum of 28 months with regimens containing paclitaxel, fluorouracil, gemcitabine, or capecitabine [123–125]. These figures need to be interpreted with caution, because selection bias is inherent in these small single-armed studies.

Inhibition of epidermal growth factor receptor (EGFR) and VEGF has shown clinical efficacy in patients with platinum-refractory disease. In a phase 2 study of cetuximab (monoclonal antibody against EGFR) in heavily pretreated patients with stage IVC NPC, measurable responses were recorded in 12% of individuals, with 48% showing stable disease [126]. The studies of pazopanib and sunitinib showed a median time to progression of 4.4 months, which is better than that for the cetuximab plus carboplatin regimen (2.7 months) [127, 128]. Moreover, the median time to progression of 5–7.5 months was shown in phase II studies of gemcitabine or capecitabine alone in similar populations [129–131]. However, in the study of sunitinib, the high incidence of hemorrhage from the upper digestive tract in NPC patients who received prior high-dose RT to the region raised many concerns.

Many immunotherapeutic strategies that are directed against EBV failed to show consistent clinical benefit. The type II EBV latency in NPC hampers the antigen presentation system and secretes immunosuppressive factors that could inhibit CD8^+^ T cell attack to NPC cells. Recently, immune checkpoints and immunotherapy have been extensively studied in an attempt to redirect host anti-tumor responses to cancer cells. Programmed death-1 (PD-1) is an immune checkpoint on the surface of T lymphocytes. The corresponding ligand called programed death-ligand 1 (PD-L1) is moderately to strongly expressed in various types of cancer, including melanoma, non-small cell lung cancer, and head and neck cancers. Fang et al. reported induction of PD-L1 by LMP1 [132]. Actually, PD-L1 is expressed in up to 90% of NPC tumors [133]. The higher expression rate of PD-1 in intratumoral CD8^+^ cells correlates with a poor prognosis in terms of overall survival, disease-free survival, and locoregional recurrence-free survival in NPC patients [134].

Pembrolizumab, which is a humanized monoclonal antibody against PD-1, was investigated in a phase 1b study [135]. Twenty-seven patients were treated with pembrolizumab. This preliminary result showed that overall response rate was 25.9% (95% CI 11.1–46.3). The optimal partnership of radiation and immunotherapy from preclinical study supports the combination of immunotherapy with RT in NPC patients [136]. Immunotherapy targeting the PD-L1 pathway should be validated in prospective randomized studies.

## Conclusion

Over the past decades, combination chemotherapy for recurrent or metastatic NPC has led to the increase in response rates. We have indeed entered the era of targeted therapy and our increasing understanding of novel systemic therapeutics has given us new insights into the management of this challenging disease in the recurrent and metastatic setting. However, more well-designed studies for treatment strategies and biomarker analysis, molecular targeted therapies, and immunotherapies are needed.
